# Genome-wide analyses identify novel risk loci for cluster headache in Han Chinese residing in Taiwan

**DOI:** 10.1186/s10194-022-01517-6

**Published:** 2022-11-21

**Authors:** Shih-Pin Chen, Chia-Lin Hsu, Yen-Feng Wang, Fu-Chi Yang, Ting-Huei Chen, Jia-Hsin Huang, Li-Ling Hope Pan, Jong-Ling Fuh, Hsueh-Chen Chang, Yi-Lun Lee, Hung-Ching Chang, Ko-Han Lee, Yu-Chuan Chang, Cathy Shen-Jang Fann, Shuu-Jiun Wang

**Affiliations:** 1 Department of Neurology, Neurological Institute, Taipei Veterans General Hospital, No. 201, Sec. 2, Shih-Pai Road, Taipei, 112 Taiwan; 2 Brain Research Center, National Yang Ming Chiao Tung University, Taipei, Taiwan; 3 Division of Translational Research, Department of Medical Research, Taipei Veterans General Hospital, Taipei, Taiwan; 4 Institute of Clinical Medicine, College of Medicine, National Yang Ming Chiao Tung University, Taipei, Taiwan; 5 School of Medicine, National Yang Ming Chiao Tung University, Taipei, Taiwan; 6 Institute of Biomedical Sciences, Academia Sinica, Taipei, Taiwan; 7 Department of Neurology, Tri-Service General Hospital, Taipei, Taiwan; 8 Department of Mathematics & Statistics, Laval University, Quebec City, QC Canada; 9 Cervo Brain Research Centre, Quebec City, QC Canada; 10 Taiwan AI Labs, Taipei, Taiwan

**Keywords:** Cluster headache, Genome-wide association study, *CAPN2*, *MERTK*, *STAB2*

## Abstract

**Background:**

Cluster headache is a highly debilitating neurological disorder with considerable inter-ethnic differences. Genome-wide association studies (GWAS) recently identified replicable genomic loci for cluster headache in Europeans, but the genetic underpinnings for cluster headache in Asians remain unclear. The objective of this study is to investigate the genetic architecture and susceptibility loci of cluster headache in Han Chinese resided in Taiwan.

**Methods:**

We conducted a two-stage genome-wide association study in a Taiwanese cohort enrolled from 2007 through 2022 to identify the genetic variants associated with cluster headache. Diagnosis of cluster headache was retrospectively ascertained with the criteria of International Classification of Headache Disorders, third edition.

Control subjects were enrolled from the Taiwan Biobank. Genotyping was conducted with the Axiom Genome-Wide Array TWB chip, followed by whole genome imputation. A polygenic risk score was developed to differentiate patients from controls. Downstream analyses including gene-set and tissue enrichment, linkage disequilibrium score regression, and pathway analyses were performed.

**Results:**

We enrolled 734 patients with cluster headache and 9,846 population-based controls. We identified three replicable loci, with the lead SNPs being rs1556780 in *CAPN2* (odds ratio = 1.59, 95% CI 1.42‒1.78, *p* = 7.61 × 10^–16^), rs10188640 in *MERTK* (odds ratio = 1.52, 95% CI 1.33‒1.73, *p* = 8.58 × 10^–13^), and rs13028839 in *STAB2* (odds ratio = 0.63, 95% CI 0.52‒0.78, *p* = 2.81 × 10^–8^), with the latter two replicating the findings in European populations. Several previously reported genes also showed significant associations with cluster headache in our samples. Polygenic risk score differentiated patients from controls with an area under the receiver operating characteristic curve of 0.77. Downstream analyses implicated circadian regulation and immunological processes in the pathogenesis of cluster headache.

**Conclusions:**

This study revealed the genetic architecture and novel susceptible loci of cluster headache in Han Chinese residing in Taiwan. Our findings support the common genetic contributions of cluster headache across ethnicities and provide novel mechanistic insights into the pathogenesis of cluster headache.

**Supplementary Information:**

The online version contains supplementary material available at 10.1186/s10194-022-01517-6.

## Background

Cluster headache is one of the most painful disorders in the world, with a prevalence of around 0.1% and a clear male predominance [1]. It is characterized by excruciating unilateral orbital, supraorbital or temporal pain attacks, with accompanying cranial autonomic symptoms or restlessness/agitation. The attacks last between 15 and 180 min but may occur up to 8 times per day and take weeks to months to remit. In some patients, the cluster headache presents as a chronic form, with attacks occurring for > 1 year without remission, or with remission periods lasting < 3 months [2]. The clinical features of cluster headache in Asian populations differ from those in Western populations, including a lower prevalence of chronic cluster headache, higher male/female ratio, and lower frequencies of restlessness or aura [3, 4]. Mechanisms underlying these ethnic differences remain unclear.

Despite the tremendous impact on the sufferers, detailed pathogenesis underlying cluster headache remains enigmatic. Because cluster headache has significant familial aggregation, attempts have been undertaken to determine its underlying genetic architectures. Hypothesis-driven approaches using candidate gene association studies, however, have not identified replicable signals. The first genome-wide association study (GWAS) investigating 99 Italian patients with cluster reported suggestive associations with genetic variants in *ADCYAP1R1 (ADCYAP receptor type I)* and *MME (membrane metalloendopeptidase)* [5]; however, these findings were not replicated subsequently. Two recent studies provided the first evidence to demonstrate genome-wide significant variants contributing to the predisposition of cluster headache in European cohorts [6, 7]. The Dutch and Norwegian study identified rs11579212 near *RP11-815 M8.1*, rs6541998 near *MERTK (MER Proto-Oncogene, Tyrosine Kinase)*, rs10184573 near *AC093590.1*, and rs2499799 near *UFL1 (UFM1 specific ligase 1)/FHL5 (four and a half LIM domains 5)*[6] whereas the combined United Kingdom (UK) and Swedish cohorts identified rs113658130 near *LINC01877/SATB2 (SATB homeobox 2)*, rs4519530 in *MERTK*, rs12121134 near *LINC01705/DUSP10* (*Dual Specificity Phosphatase 10*), and rs11153082 in *FHL5* to be associated with cluster headache [7]. Considering the inter-ethnic variability of clinical characteristics [3], it is uncertain whether these novel loci are replicable in other populations.

To interrogate the genetic architecture of cluster headache in Asians, we performed a two-stage GWAS in a total of 734 clinic-based patients and 9,846 population-based controls. We also established polygenic risk score (PRS) models to differentiate patients from controls and conducted downstream analyses to investigate genes and potential pathogenic mechanisms of cluster headache.

## Methods

### Study participants and clinical evaluations

This study was a two-stage case–control GWAS, including a discovery cohort and a replication cohort, followed by a combined analysis of both cohorts. All participants were unrelated and of Han Chinese ancestry. Patients with cluster headache were enrolled from the headache or neurological clinics of TVGH and TSGH, Taiwan, as well as the Taiwan Precision Medicine Initiative, from 2007 through 2022. Patients who were recruited after May 2012 were assigned to the discovery cohort and subjects recruited prior to that were assigned to the replication cohort. The control subjects were recruited from the Taiwan Biobank, the largest publicly available genetic database of individuals with East Asian ancestry that provides adequate coverage of genetic diversity across all Han Chinese [8]. To evaluate the genetic correlation of cluster headache and migraine, we also recruited an independent cohort of migraine patients from TVGH. The inclusion criteria for all participants include (1) age between 20 and 70 years old and (2) could fully understand the study objective and willing to provide written informed consent. Patients were eligible if they were diagnosed with cluster headache or migraine by board-certified neurologists based on the criteria proposed in the International Classification of Headache Disorders, third edition (ICHD-3) [2]. The exclusion criteria for all participants included history of major systemic illnesses or major neuropsychiatric disorders. Control subjects who have a history of moderate or severe headaches, first-degree relatives with a history of migraine or severe headaches, or any kinship relatedness with the patients were also excluded.

### Single nucleotide polymorphism (SNP) genotyping

SNP genotyping was conducted with the Affymetrix Axiom Genome-Wide TWB2 Array Plate at the National Center for Genomics Medicine, Academia Sinica, Taiwan. SNP genotypes were called using the Axiom GT1 algorithm. Quality control (QC) criteria for SNPs were applied to exclude SNPs if they (a) were monomorphic in both cases and controls, (b) had a minor allele frequency < 0.01, (c) had a total call rate < 95% in cases and controls combined, (d) showed significant (*P* < 0.00001) deviation from Hardy–Weinberg equilibrium in case or controls, or (e) a significant difference in the genotype call rates between cases and controls (*P* < 0.001). For sample filtering, we excluded arrays with generated genotypes for fewer than 95% of loci. Heterozygosity of SNPs on the X-chromosome was used to verify the sex of the samples. PLINK software (version 1.90) [9] was used to identify samples with genetic relatedness, indicating that they were from the same individual (or monozygotic twins) or from first-, second- or third-degree relatives. These determinations were made based on evidence for cryptic relatedness from identity-by-descent status (pi-hat cutoff of 0.125).

### Genotype imputation analysis

We conducted a genotype imputation analysis using the 1000 Genomes Phase 3 data [10] and TWB whole-genome sequencing data as reference and IMPUTE2 [11–13]. Well-imputed SNPs (information score > 0.8) were retained followed by systematic QC, as described above [14]. For sample filtering, we excluded arrays with generated genotypes for fewer than 95% of loci.

### Statistical analysis

Association analyses were carried out by comparing allele or genotype frequencies between cases and controls. The Manhattan Plot and quantile–quantile (Q-Q) plots were generated using the “qqman” package in R [15]. Detection of population stratification was carried out by using principal component analysis (PCA). The genomic inflation factor (GIF) was calculated and the top 10 principal components (PCs) via Eigenstrat in typing datasets. We calculated the distribution of expected *p* values under the null hypothesis and genomic inflation value (λ = 1). Logistic regression was used to evaluate association of SNPs with cluster headache by adjusting age, sex, and the top 10 PCs (PC1‒PC10), and an additive genetic model was conducted using PLINK (version 1.9) [9]. *P* < 5 × 10^–8^ was designated as GWAS significance and *p* < 5 × 10^–5^ was designated as suggestive GWAS significance. The positional gene mapping and fine mapping of significant loci were generated using LocusZoom [16] and Probabilistic Identification of Causal SNPs (PICS2) [17]. The proportion of variance explained by a given SNP was calculated using Nagelkerke pseudo R^2^.

### Gene-based analysis

We performed the Multimarker Analysis of GenoMic Annotation (MAGMA) gene-based association analysis [18] implemented in FUMA [19], which calculates a gene test-statistic (*p*-value) based on all SNPs located within genes, using imputation data.

### Previously reported cluster headache or migraine loci

Genetic loci previously reported to be associated with cluster headache were tested for association in our samples [5–7, 20–26]. We also conducted meta-analysis of these loci by combining the results from previous studies and ours with METAL [27]. As recent GWASs suggested a potential genetic correlation of cluster headache with migraine [6, 7], we also tested the association of migraine-associated loci [28, 29] in the current sample.

### Univariate LD-score regression

Linkage Disequilibrium Score Regression (LDSC v1.0.1) was used to estimate the proportion of a true polygenic signal opposed to confounding biases, and to calculate SNP-based heritability [30]. We estimated LD Scores from 1,446 samples in the Taiwan Biobank whole-genome sequencing data using an unbiased estimator of r^2^ with 1-cM windows, singletons excluded and no r^2^ cutoff. Heritability estimates were converted to the liability scale assuming a population prevalence of cluster headache of 0.1%.

### Polygenic risk score derivation

Four sets of polygenic risk scores were developed to discriminate patients from controls. In the first set, we computed genome-wide PRS derived from the whole genome imputation data following a method reported previously [31]. We used the GWAS of the discovery cohort (i.e., the training set) for generating the estimates of regression coefficients, its standard error and associated p-value (${\widehat{\beta }}_{j}$, ${\widehat{\sigma }}_{j}$ and ${p}_{j}$) for each SNP *j* based on the univariate logistic regression analysis. With SNPs that passed the QC in GWAS analysis, the SNPs with MAF less than 1% or Indels were excluded. To calculate PRS, we used the standard clumping and thresholding (C + T) method [32] using two hyperparameters for model building: the cut-off of correlation ${r}^{2}$ and p-value threshold *p.* The optimal PRS model was chosen from the pair of (${r}^{2}, p$) that maximized the prediction performance, area under the receiver-operator characteristics curve (AUC), evaluated in the testing (i.e., replication) cohort. In the second set, the PRS was derived from all the previously reported loci associated with cluster headache. The prediction performance was also evaluated with AUC. The 3^rd^ and 4^th^ sets of PRS were derived from the significant SNPs of the two previously reported European cluster headache GWASs [6, 7] respectively.

### Gene expression, tissue enrichment analysis, and pathway analysis

The tissue specific expression of the cluster headache-associated genes was explored based on the expression data from the Genotype-Tissue Expression (GTEx) project [33] using the GTEx platform or the MAGMA gene property in FUMA. We also implemented GIGSEA (Genotype Imputed Gene Set Enrichment Analysis) [34] to infer differential gene expression and interrogate gene set enrichment for the trait-associated SNPs. The gene expression levels were imputed from GWAS summary data of cluster headache by Elastic net (ENet) regression models using MetaXcan [35] or the pathway analyses of MetaXcan output, we employed the weighted linear regression model of the GIGSEA [34] with empirical p-values incorporating 1,000 permutations for Gene Ontology (GO), Kyoto Encyclopedia of Genes and Genomes (KEGG), and Reactome (REAC) [36–38]. Following the guidance of GIGSEA, the Bayes factor was used to correct the multiple hypothesis testing [39]. Stringent thresholds for significant associations were set as the empirical *p*-values < 0.05 and Bayes factor [BF] > 100 in the pathway enrichment analyses in brain tissues. Of note, we selected the enriched pathways with the gene sets that contain from 15 to 500 genes as suggested by GSEA manual.

## Results

### Characteristics of study subjects

The two-stage GWAS included a discovery cohort consisting of 359 cluster headache patients and 4786 population-based controls and a replication cohort comprised 375 patients and 5060 population-based controls. Demographic characteristics of participants are shown in Additional File 1.

### Association analysis

After applying stringent QC criteria, we obtained 549,611 SNPs with an average call rate of 99.78% ± 0.25%. After imputation, 6,630,979 loci were yielded. Q-Q plot (Additional File 2) and PCA (Additional File 3) showed no significant population stratification. In the combined analysis, the LDSC intercept was 0.9962 (SE 0.0061), indicating little inflation due to factors other than polygenic architecture. In addition, we estimated the SNP-based heritability (h^2^) of cluster headache at 16.51% (SE 4.42%) on the observed scale and 22.5% (SE 6.03%) on the liability scale.

**Fig. 1:**
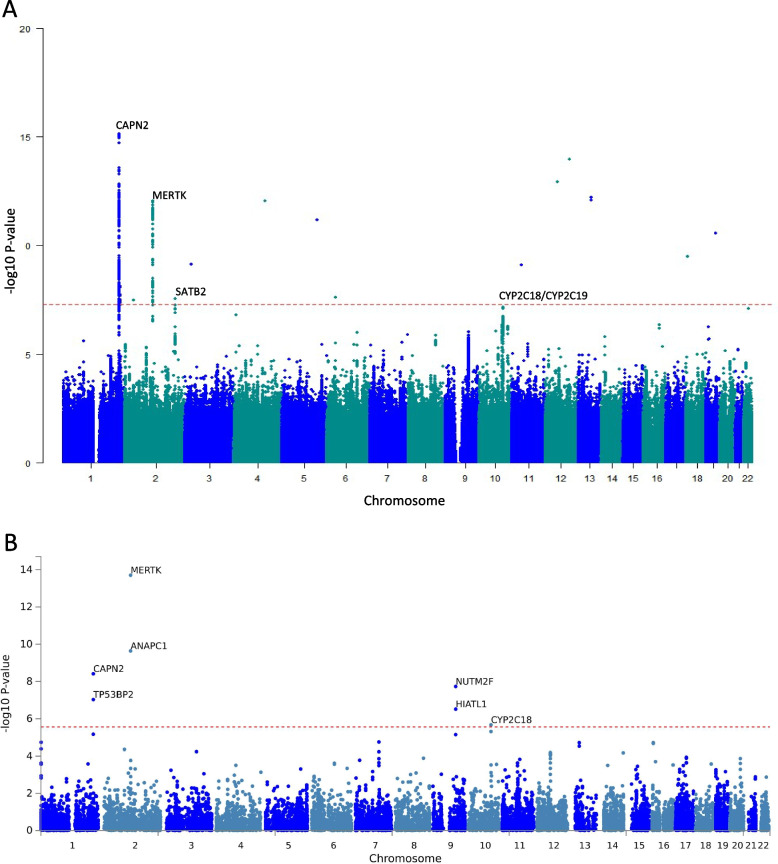
Manhattan plots for the GWAS and gene-based analysis. **A** Manhattan plot for the associations of SNPs with cluster headache. The horizontal axis shows the chromosomal position, and the vertical axis shows the significance of tested markers. The threshold for genome wide significance (*p* < 5 × 10^–8^) is indicated by a red dash line. **B** Manhattan plot of the gene-based test as computed by Multimarker Analysis of GenoMic Annotation (MAGMA). The input SNPs were mapped to 18,460 protein coding genes. The red dash line indicates the genome wide significance defined at *p* = 0.05/18,460 = 2.709 × 10^–6^

**Fig. 2:**
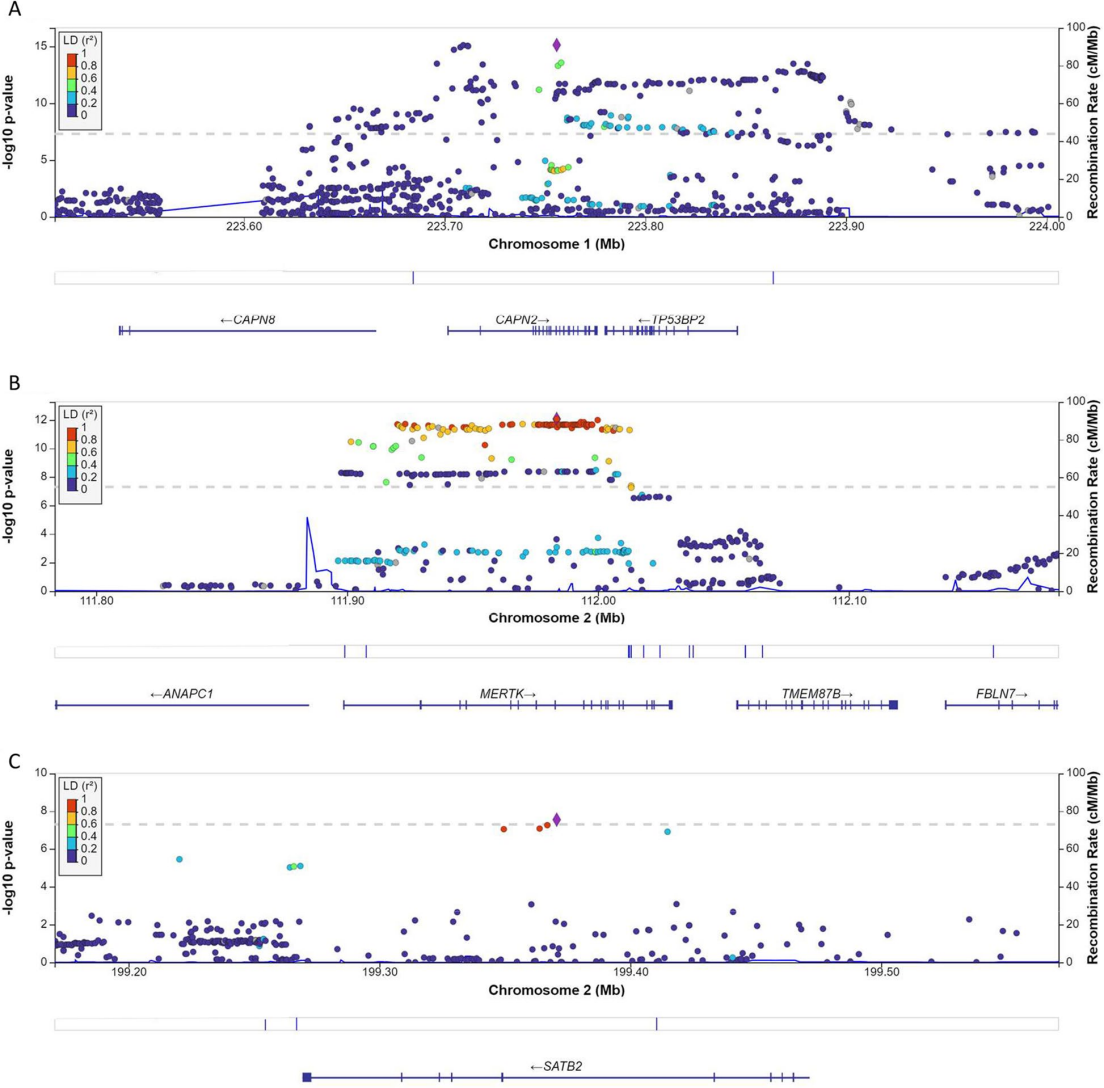
Regional association plots of the two genome-wide significant cluster headache loci (**A**) *CAPN2*, (**B**) *MERTK*, and (**C**) *SATB2.* Each dot represents a single nucleotide polymorphism (SNP) derived from the fine-mapped imputation data. The horizontal axis gives the genomic coordinate and the vertical axis the significance level (-log_10_ *p* value). The top SNP for each locus is marked with a purple diamond (CRCh38/hg19). SNPs are colored based on their correlation (r^2^) with the labeled lead SNP according to the legend. The solid blue line shows the recombination rate from 1000 Genomes EAS data (right vertical axis). The gray dash line corresponds to *p* = 5 × 10^−8^. Figures were obtained from LocusZoom

The two-stage genome-wide analysis using imputation data identified one replicable genomic locus in *Calpain 2* (*CAPN2*) with multiple SNPs reaching GWAS significance at both discovery and replication cohorts (Fig. 1A and Additional File 4). Combined analysis found additional two loci with GWAS significance in *MERTK and SATB2* (Table 1) and one locus with suggestive GWAS significance in *CYP2C18*/*CYP2C19* (Fig. 1A). Of note, indels or singleton SNPs without significant LD with other SNPs that were deemed spurious association signals. The regional association plots for the significant loci were demonstrated in Fig. 2. In addition, fine mapping with PICS identified multiple variants with causal probability larger than 0.2, but only rs4653500 (PICS probability = 0.46) has eQTL on *CANP2*.

**Table 1:** Basic demographics of participants

Characteristics	N	Mean age ± SD (yr)	Male, n (%)	*P value*
***Cluster headache GWAS***				
**Discovery cohort**				
Cluster headache	359	36.39 ± 10.58	293 (81.62%)	0.335
Controls	4,786	42.98 ± 7.56	4,000 (83.58%)	
**Replication cohort**				
Cluster headache	375	36.74 ± 10.61	298 (79.47%)	0.159
Controls	5,060	42.84 ± 7.58	4,012 (79.29%)	
**Combined analysis**				
Cluster headache	734	36.57 ± 10.59	591 (80.52%)	0.566
Controls	9,846	42.91 ± 7.57	8,012 (81.37%)	
***Migraine cohort for computation of genetic correlation with cluster headache***				
Migraineurs	3,173	38.51 ± 11.77	638 (20.11%)	< 0.001
Controls	24,528	43.31 ± 7.55	8,013 (32.67%)	

*P p* value for sex ratio (by chi-square test)

### Gene-based association testing

Gene-based association testing used the mean association signal from all SNPs within each gene, accounting for LD. Seven genes passed the threshold of statistical significance: *MERTK*, *ANAPC1* (*anaphase promoting complex subunit 1*), *CAPN2*, *NUTM2F (NUT Family Member 2F)*, *TP53BP2 (Tumor Protein P53 Binding Protein 2)*, *HIATL1 (Hippocampus Abundant Transcript-Like Protein 1)*, and *CYP2C18* (Fig. 1B). The Q-Q plot of the gene-based test computed by MAGMA was shown in Additional File 5.

### Previously reported cluster headache loci

Among the previously reported cluster headache-associated SNPs, rs4519530 in *MERTK* and rs6541998 near *MERTK* were successfully replicated in our sample using imputation data. Some SNPs were unavailable or insignificant in our sample, but the lead SNPs within or near the same genes were found to have suggestive GWAS significance (Table 2). Most of the variants, particularly those in *MERTK* and *FHL5*, were more significant in meta-analysis (Table 2); however, substantial heterogeneity was noted in some of them (Additional File 6).

**Table 2:** The Association of previously reported cluster headache loci in the current sample

Chr	Gene	Index SNP	Risk allele	Con^a^	OR [95%CI]	P1^b^	P2^c^	Ref No
1	*near RP11-815M8.1*	rs11579212	C	2	0.97 [0.78–1.2]	0.30	0.09	6
1	*LINC01705/DUSP10*	rs6687758	G	1	1.07 [0.94–1.23]	0.23	1.58 × 10^–3^	7
1	*LINC01705/DUSP10*	rs12121134	T	2	0.61 [0.22–1.69]	0.21	5.23 × 10^–3^	7
1	*LINC01705*	rs545037820	G	3	0.75 [0.50–1.12]	0.02		
1	*near LINC01705*	rs2034485	T	3	1.31 [1.16–1.49]	1.16 × 10^–6^		
1	*DUSP10*	rs145531779	C	3	1.43 [1.11–1.83]	0.02		
2	*MERTK*	rs4519530	C	2	1.50 [1.32–1.72]	4.69 × 10^–12^	9.30 × 10^–27^	7
2	*near MERTK*	rs6541998	C	2	1.43 [1.25–1.63]	1.86 × 10^–8^	6.23 × 10^–15^	6
2	*MERTK*	rs10188640 rs10188642	A	3	1.52 [1.33–1.73]	8.58 × 10^–13^		
2	*near LINC01877/SATB2*	rs4675692 rs113658130	G C	NA				7
2	*LINC01877*	rs13394614	G	3	1.34 [1.18–1.52]	5.42 × 10^–7^		
2	*STAB2*	rs13028839	A	3	0.63 [0.52–0.78]	2.81 × 10^–8^		
3	*MME*	rs147564881	C	NA				5
3	*MME*	rs56208271	A	3	1.61 [1.17–2.22]	4.56 × 10^–3^		
3	*near MME*	rs143162143	C	3	2.29 [1.56–3.36]	1.15 × 10^–4^		
4	*CLOCK*	rs12649507	G	2	0.88 [0.78–1.01]	0.07	1.02 × 10^–3^	21
4	*CLOCK*	rs369023808	G	3	1.19 [0.76–1.88]	0.04		
4	*near CLOCK*	rs34945396	C	3	0.78 [0.66–0.92]	5.09 × 10^–3^		
4	*ADH4*	rs1126671	T	1	1.72 [0.60–4.93]	0.04	1.46 × 10^–3^	20
4	*ADH4*	rs56314548	C	3	0.77 [0.51–1.17]	0.58		
6	*HCRTR2*	rs3800539	A	2	1.13 [0.99–1.29]	0.14	0.13	26
6	*HCRTR2*	rs10498801	A	2	0.88 [0.75–1.02]	0.03	0.34	26
6	*HCRTR2*	rs2653349	A	2	0.77 [0.57–1.03]	0.49	0.10	23, 24
6	*HCRTR2*	rs3122156	G	NA				25
6	*HCRTR2*	rs78769350	G	3	0.69 [0.46–1.06]	0.03		
6	*near UFL1/FHL5*	rs2499799	T	2	1.08 [0.85–1.36]	0.74	4.00 × 10^–3^	6
6	*FHL5*	rs11153082	G	2	1.19 [1.04–1.35]	1.99 × 10^–4^	7.54 × 10^–11^	7
6	*FHL5*	rs9386670	A	2	1.17 [1.02–1.34]	9.72 × 10^–4^	1.09 × 10^–9^	7
6	*UFL1*	rs2255552	G	3	1.18 [1.04–1.35]	1.83 × 10^–4^		
6	*FHL5*	rs57425973	A	3	1.2 [1.05–1.37]	6.21 × 10^–5^		
7	*ADCYAP1R1*	rs12668955	A	1	1.07 [0.96–1.19]	0.22	1.7 × 10^–4^	5
7	*ADCYAP1R1*	rs201504906 rs200124908	C	3	1.72 [1.31–2.26]	1.01 × 10^–5^		
12	*CRY1*	rs8192440	A	1	0.96 [0.66–1.41]	0.42	0.09438	22

*Abbreviations*: *Chr* Chromosome, *SNP* Single nucleotide polymorphism, *Con* Condition, *OR* Odds ratio, *CI* Confidence interval, Risk allele, allele with higher frequency in cases compared to controls, *NA* Not available

^a^Condition: 1. Information obtained from real genotyping data. 2. Information derived from imputation data. 3. Most significant SNP within or near the same gene in the imputation data

^b^The *p* values of Cochran-Armitage Trend Test

^c^The *p* values of meta-analysis of the previous studies and the current study

### Polygenic risk score

**Fig. 3:**
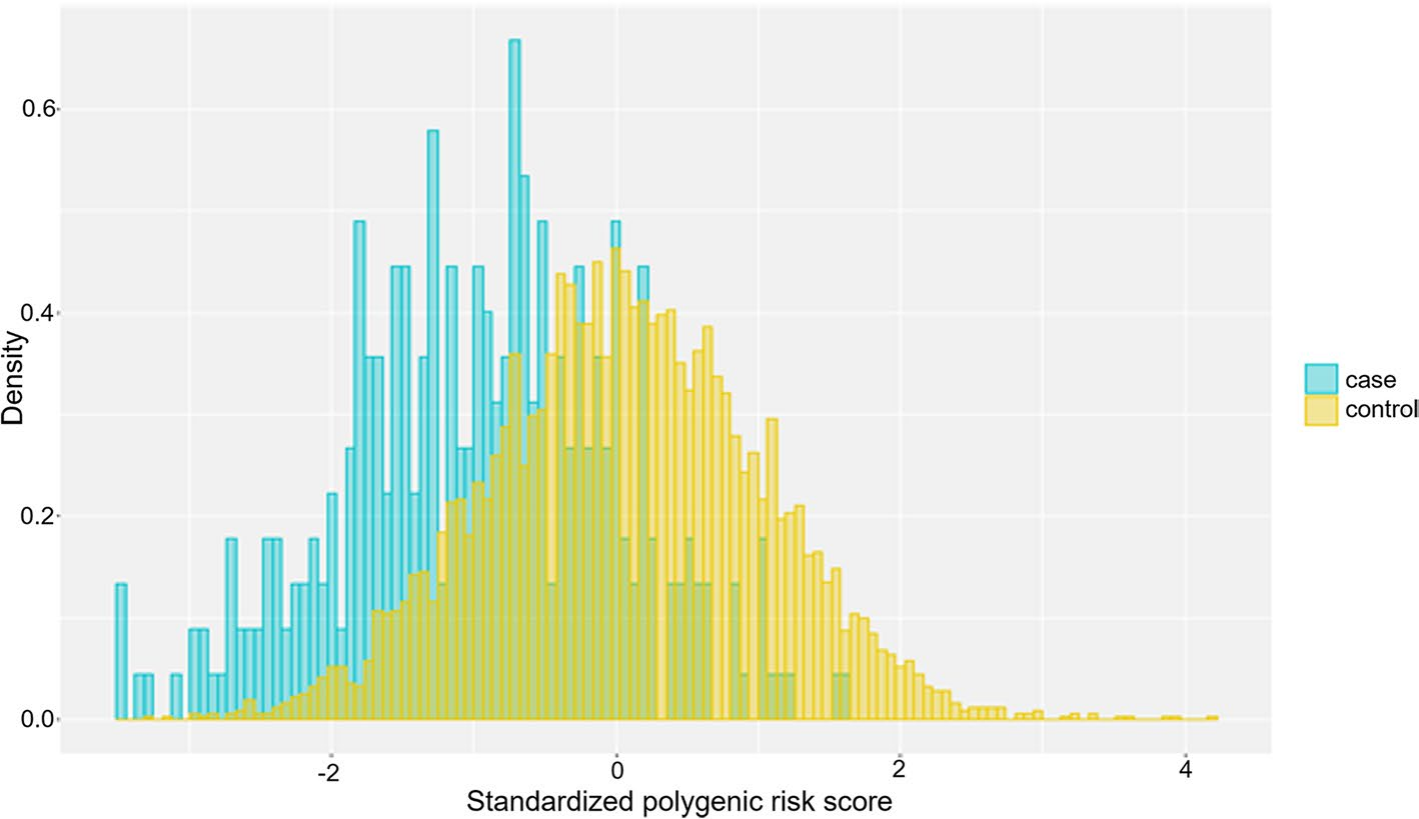
Genome-wide polygenic risk score (PRS) for cluster headache. The density distributions of polygenic score for cluster headache in testing dataset. The x-axis represents polygenic score, with values scaled to a mean of 0 and standard deviation of 1. Cases: blue; Controls: yellow

We calculated two sets of PRS. In the first set using the C + T method for genome-wide data, the distribution of the PRS for cluster headache in testing dataset was shown in Fig. 3. Grid search found that the PRS consisted of SNPs with *r*^*2*^ = 0.1 and *p*-value = 0.05 was the best model to differentiate patients from controls (AUC = 0.772, 95% confidence interval (CI) 0.747–0.797). The SNPs within the PRS model had a combined explained variance (i.e., Nagelkerke pseudo R^2^) of 16.97%. In the second set which used only the previously reported cluster headache loci (listed in Table 3) to derive PRS, the AUC was 0.633 (95% CI 0.604–0.662). The 3^rd^ set of PRS derived from the Dutch and Norwegian study [6] had an AUC of 0.536 [0.504–0.567] while the 4^th^ set from UK and Swedish study [7] had an AUC of 0.583 [0.553–0.613].

**Table 3:** Lead SNPs of GWAS significant loci in the combined analysis for patients with cluster headache in Taiwan

SNP	Chr	Position	Gene	Minor allele	Stage	MAF(Case, Control)	OR	95% CI	*P* value^a^	eQTL mapped genes^c^
rs1556780	1	223,755,834	*CAPN2*	G	1	(0.354, 0.253)	1.637	1.390‒1.927	2.34 × 10^–9^	*CAPN2*
					2	(0.344, 0.253)	1.540	1.316‒1.801	5.31 × 10^–8^	
					Com	(0.329, 0.253)	1.586	1.416‒1.775	7.61 × 10^–16^	
rs10188640	2	111,983,520	*MERTK*	A	1	(0.345, 0.257)	1.522	1.297‒1.787	2.17 × 10^–7^	*AC012442.5*, *FBLN7*, *MERTK*, *RGPD8*, *TMEM87B*, *TTL*, *ZC3H8*
					2	(0.343, 0.260)	1.481	1.266 ‒1.733	8.05 × 10^–7^	
					Com	(0.349, 0.258)	1.501	1.342‒1.679	1.25 × 10^–12^	
rs13028839	2	199,370,629	*SATB2*	A	1	(0.096, 0.154)	0.577	0.447–0.746	2.02 × 10^–5^	*C2orf69*, *SATB2*, *AC019330.1*
					2	(0.099, 0.149)	0.639	0.493–0.829	3.46 × 10^–4^	
					Com	(0.102, 0.151)	0.634	0.518‒0.778	2.81 × 10^–8^	

*Abbreviations*: *SNP* Single nucleotide polymorphism, *Chr* Chromosome, *OR* Odds ratio, *CI* Confidence interval, *MAF* Minor allele frequency

^a^The *p* values of Cochran-Armitage Trend Test The eQTL mapping was done with GTEx v.8

### Genetic correlation between cluster headache and migraine

We recruited a cohort consisting of 3,173 Taiwanese migraine patients and 24,528 controls and estimated the genetic covariance between cluster headache and migraine on the liability scale was 21.0% (SE 5.7%) and the genetic correlation was 42.91% (SE 16.31%, *P* = 0.009). We have also examined 125 migraine-associated loci reported in previous migraine GWAS in the current sample of cluster headache.^27, 28^ Some SNPs or variants with high LD within or near the migraine-associated loci were found to have suggestive GWAS significance in the current cluster headache dataset (Table 4).

**Table 4:** Previously reported migraine loci with suggestive GWAS significance in the current sample

Chr	Gene	Index SNP	Risk allele	Con^a^	OR [95%CI]	*P* value^b^
2	*MYO3B*	rs6753065	G	2	1.43 [1.20–1.71]	4.91 × 10^–5^
6	near *GJA1*	rs28455731	T	1	1.32 [1.15–1.50]	4.03 × 10^–5^
6	near *GJA1*	rs9482172	C	2	1.33 [1.17–1.52]	1.98 × 10^–5^
6	near *PCMT1*	rs560489087	T	2	2.12 [1.49–3.03]	2.05 × 10^–5^
9	*NFIB*	rs10756554	T	2	0.79 [0.71–0.89]	3.33 × 10^–5^
10	*PLCE1*	rs4244285	A	1	1.34 [1.20–1.49]	1.76 × 10^–7^
10	*PLCE1*	rs12571421	G	2	1.34 [1.20–1.49]	1.88 × 10^–7^
14	near *ITPK1*	rs150587675	A	1	1.68 [1.32–2.14]	2.23 × 10^–5^
17	*HOXB3*	rs3214184	TTAACA^c^	2	1.33 [1.16–1.51]	2.36 × 10^–5^
18	near *SKOR2*	rs62095427	A	2	0.62 [0.49–0.76]	9.95 × 10^–6^

*Abbreviations*: *Chr* Chromosome, *SNP* Single nucleotide polymorphism, *Con* Condition, *OR* Odds ratio, *CI* Confidence interval, Risk allele, allele with higher frequency in cases compared to controls, *NA* Not available

^a^Condition: 1. Information obtained from real genotyping data. 2. Most significant SNP within or near the same gene in the imputation data

^b^The *p* values of Cochran-Armitage Trend Test

^c^The variant is an INDEL (T/TTAACA)

### eQTL analysis

eQTL analysis using the GTEx online platform [33] (accessed on June 20, 2022) found that the lead SNP rs1556780 in *CAPN2* and rs10188640 in *MERTK* had significant eQTLs on several genes (Table 1). With MetaXcan to infer the correlation between genetic variants and gene expression, we found that the genetic variants in *MERTK* were significantly associated with the gene expression in the pituitary gland and caudate (basal ganglia) with a false discovery rate (FDR) of less than 0.001 (Additional File 7).

### Functional enrichment and pathway analysis

MAGMA gene-property analyses implemented in FUMA did not demonstrate significantly enriched tissues. GIGSEA biological pathway enrichment analyses based on the MetaXcan gene-level summary statistics found that the pathways were predominantly enriched in the pituitary gland, hippocampus, cerebellum, and basal ganglion. Top significantly associated pathways in these tissues that may be relevant to cluster headache include pathways involved in synaptic transmission, transmembrane protein kinase activity, immune responses, mitochondrial function, and metalloendopeptidase activity (Additional File 8 and Additional File 9).

## Discussion

We identified three susceptibility loci, in *CAPN2, MERTK*, and SATB2, as well as one suggestive locus at *CYP2C18*/*CYP2C19* at genome-wide level in patients with cluster headache in Taiwan. To the best of our knowledge, this is the first GWAS of cluster headache performed in Han Chinese and the first in Asians. While replicating the susceptibility genes recently identified in GWASs in patients of European ancestries [6, 7] suggested the validity of our study, we also identified novel genes implicating potential inter-ethnic differences. The association effect sizes are relatively large and similar to those observed in European GWASs [6, 7]. These results suggest that some phenotypes of cluster headache might be driven by these selected loci with large effect size, although further studies are needed to explore the genotype–phenotype association. In addition, several other risk loci identified in previous studies are of suggestive GWAS significance in our samples, suggesting that future studies with larger sample sizes might validate the associations of these loci with cluster headache. Moreover, the superior discriminative capacity of genome-wide PRS than the PRS composed of known cluster headache-associated loci suggests that additional loci with smaller effect sizes might also contribute to the genetic basis of cluster headache. Nevertheless, the clinical utility of PRS remains to be explored.

The novel gene *CAPN2* identified in our study encodes calpain 2, a calcium-regulated non-lysosomal thiol-protease involved in cytoskeletal remodeling and signal transduction. Calpain 1/2 have been known to mediate Ca^2+^ influx and mediate degradation of suprachiasmatic nucleus (SCN) circadian oscillatory protein (SCOP) [40] in SCN neurons, which may contribute to coordinated regulation of circadian rhythms. In addition, calpain 1/2 play opposite roles in retinal ganglion cell (RGC) degeneration induced by ischemia/reperfusion injury [41], while degeneration of RGCs could lead to impaired circadian rhythmicity. Another potential implication of *CAPN2* may be that the most commonly used preventive drug for cluster headache, the L-type calcium channel blocker verapamil, has been known to abrogate calpain activation [42]. Furthermore, calpain was found to mediate capsaicin-induced ablation of transient receptor potential vanilloid subtype 1 (TRPV1)-positive trigeminal afferent terminals [43], which may mediate the release of calcitonin gene-related peptide (CGRP) and contribute to the pathogenesis of cluster headache.

We successfully replicated *MERTK* and *SATB2* identified in previous cluster headache GWASs [6, 7]. *MERTK* encodes a protein belongs to the MER/AXL/TYRO3 receptor kinase family. In addition to regulating microglia-mediated neuroinflammation and astrocyte-mediated neuronal synaptic remodeling proposed in previous studies [6, 7]. *MERTK* functions in the retinal pigment epithelium as a regulator of photoreceptor phagocytosis, which is a circadian-regulated process indispensable for vision [44]. Mutations of *MERTK* cause degeneration of photoreceptors, which in turn lead to the loss of photic and circadian control and reduced production of melanopsin mRNA in RGCs [45]. As melanopsin-expressing RGCs are responsible for circadian photoreception and project to SCN and hypothalamus [46], *MERTK* may thus indirectly participate in the pathogenesis of cluster headache. In addition, enrichment analysis showed significant expression of *MERTK* in pituitary gland, which could potentially contribute to the altered hormonal expression in cluster headache [47]. *SATB2* encodes a DNA binding protein that specifically binds nuclear matrix attachment regions and involves in transcription regulation and chromatin remodeling. Previous GWAS suggested that it may be associated with hypothalamic dopaminergic neurons and structures responsible for nociceptive processing [6, 7]. *SATB2* was also known as an important transcription factor of RGCs in primates [48]. In addition, both GWAS and gene-based association analysis suggested *CYP2C18* as a potential novel susceptibility gene for cluster headache. Interesting, CYP2C18 is a cytochrome P450 monooxygenase involved in retinoid metabolism [49], while retinoic acid is a molecular trigger of RGC hyperactivity [50]. Taken together, the top implicated genes in our study may modulate the function of RGCs; however, how this could be involved in cluster headache pathogenesis particularly circadian rhythmicity requires further validation.

Gene-based association testing also identified several possible additional loci, among these, *ANAPC1* has also been identified in another cluster headache GWAS using gene-based analysis, with enriched expression in the brain, particularly neurons [7]. In addition, among the genes that may be potentially affected by variants in *MERTK*, i.e., with significant eQTL expression, *TMEM87B* (*transmembrane protein 87B*) and *FBLN7* (*Fibulin 7*) have been identified in prior GWASs via gene-based analysis or eQTL analysis [6, 7], suggesting their involvement in cluster headache. Moreover, we found evidence of suggestive association among previously suggested loci to be involved in cluster headache, including *LINC01877, LINC01705, ADCYAP1R1*, *and MME*. Although these genes are functionally plausible for the pathogenesis of cluster headache, future studies with larger sample sizes are needed to validate these findings.

We found considerable heritability of cluster headache and migraine and significant genetic correlation between these two primary headache disorders, which corroborates with the clinical observations that both disorders exhibit features of trigeminovascular activation and respond to similar treatments such as triptans or CGRP monoclonal antibodies. Some previously reported migraine loci were also found to have suggestive GWAS significance in our current sample, which may contribute to the share biology between migraine and cluster headache. In addition, pathway analyses suggested that biological processes associated with synaptic transmission or immune responses may be involved in the pathogenesis of cluster headache. In fact, immunological processes have long been considered important in the pathogenesis of migraine and we have recently found *HLA* class I alleles are associated with clinic-based migraine [51].

Our study has limitations. First, the sample size is relatively small. However, the successful validation of the findings of previous GWASs and replicable signals in both stages of our GWAS after stringent quality control suggest that our findings are unlikely spurious. Second, the identified variants might not be the true causal variants and it remains unknown whether the expression of the implicated genes is truly altered in patients even though eQTL analysis suggested that these variants could affect gene expression in certain tissues. Finally, although the implicated genes were plausibly relevant to the pathogenesis of cluster headache in in silico functional analysis, we were unable to validate the function of these genes in vivo at the current stage owing to the limitation of the availability of biological samples such as brain tissues or retina from the patients. Further in-depth functional analysis at molecular level, at least in a subset of the patients, are needed the increase the credibility of the findings..

## Conclusions

In conclusion, our data provide the genetic architecture and mechanistic insights into cluster headache, particularly in Asians, which has been under-represented in previous genetic studies of cluster headache. Future GWAS with patients from multiple ethnicities are required to identify shared and independent genetic determinants of cluster headache across different populations and to better understand the molecular mechanisms of this debilitating disorder.

## Supplementary Information

**Additional file 1: Supplementary Table 1.** Demographics and of headache characteristics in patients with cluster headache.

**Additional file 2:** **Supplemental Figure 1.** Quantile-quantile plot of the GWAS results of cluster headache.

**Additional file 3:** **Supplemental Figure 2.** Principal component analysis (PCA) plot. The horizontal and vertical axes are the first and second dimensions from principal component analysis based on the cluster headache GWAS samples. Blue dots indicate controls and red dots indicate patients.

**Additional file 4:** **Supplemental Figure 3.** Manhattan plot of the discovery cohort (A) and replication cohort (B).

**Additional file 5:** **Supplemental Figure 4.** Q-Q plot of the gene-based test computed by MAGMA. The horizontal axis shows -log10 p values expected under the null distribution. The vertical axis shows observed -log10 p values.

**Additional file 6:** **Supplemental Table 2.** Meta-analysis of previous studies and the current study for previously reported cluster-associated loci.

**Additional file 7:** **Supplemental Figure 5.** eQTL analysis by MetaXcan

**Additional file 8:** **Supplemental Table 3**. GIGSEA Biological Pathway Enrichment in brain tissues. The significant level of empirical *P*-value was 0.05 and the BayesFactor was 100. Top significantly associated pathways in these tissues that may be relevant to cluster headache were listed in the table. UsedGenes indicates number of gene used in the enrichment estimation of the GIGSEA model.

**Additional file 9:** **Supplemental Figure 6.** GIGSEA pathway analysis of the genes relevant to cluster headache GWAS variants.

## Data Availability

Data described in the manuscript, code book, and analytic code, intended for reasonable use, will be made available upon request by contacting the corresponding authors. Anonymized data from TWB is available for application by contacting biobank@gate.sinica.edu.tw.

## Abbreviations

ADCYAP1R1: ADCYAP receptor type I
ANAPC1: Anaphase promoting complex subunit 1
CAPN2: Calpain 2
CGRP: Calcitonin gene-related peptide
DUSP10: Dual Specificity Phosphatase 10
FHL5: Four and a half LIM domains 5
FBLN7: Fibulin 7
GWAS: Genome-wide association study
HIATL1: Hippocampus Abundant Transcript-Like Protein 1
ICHD-3: International classification of headache disorders, third edition
MME: Membrane metalloendopeptidase
MERTK: MER proto-oncogene, tyrosine kinase
PRS: Polygenic risk score
NUTM2F: NUT Family Member 2F
SATB2: SATB homeobox 2
SNP: Single nucleotide polymorphism
TMEM87B: Transmembrane protein 87B
TP53BP2: Tumor protein P53 binding protein 2
TRPV1: Transient receptor potential vanilloid subtype 1
UFL1: UFM1 specific ligase 1
