# Supplementary material for: Genome-wide analyses identify novel risk loci for cluster headache in Han Chinese residing in Taiwan
Source: J Headache Pain. 2022 Nov 21;23(1):147. doi: 10.1186/s10194-022-01517-6 (PMC9677903; doi:10.1186/s10194-022-01517-6)
Supplement: Supplementary file 1 — Additional file 1: Supplementary Table 1. Demographics and of headache characteristics in patients with cluster headache. [file 10194_2022_1517_MOESM1_ESM.docx]

**Supplementary Table 1. Demographics and of headache characteristics in patients with cluster headache.**

| **Category** |  |
| --- | --- |
| **Demographics** |  |
| Height (cm) | 171.2 (7.3) |
| Weight (kg) | 70.2 (12.5) |
| Chronic cluster headache, N (%) | 12 (1.6%) |
| Onset age (y/o) | 25.4 (9.9) |
| **Personal and family history (%)** |  |
| Current smoker | 46.3% |
| Ex-smoker | 12.0% |
| Non-smoker | 41.5% |
| Drinking rate | 81.8% |
| Head injury with LOC | 6.3% |
| Accompanied with migraine | 15.3% |
| Family history of migraine | 24.4% |
| Family history of cluster headache | 3.7% |
| **Headache profiles** |  |
| Headache severity (NRS) | 9.3 (1.2) |
| Mean attack frequency (N/day) | 2.6 (1.5) |
| Mean attack duration (minutes) | 113.6 (77.4) |
| Mean bout duration (weeks) | 6.9 (8.4) |
| Mean interbout duration (months) | 19.0 (23.1) |
| Mean bout frequency (year) | 1.5 (3.5) |
| Headache Laterality |  |
| Side locked (right) | 53.0% |
| Side locked (left) | 34.5% |
| Side shifting | 12.5% |
| Headache location |  |
| Temporal region | 82.4% |
| Orbital/retro-orbital region | 78.0% |
| Headache periodicity |  |
| With circannual periodicity | 86.4% |
| With circadian periodicity | 74.4% |
| With nocturnal attacks | 44.7% |

*Data is presented as mean (SD) or % as appropriate. LOC: Loss of consciousness; NRS: numeric rating scale.
