# Supplementary material for: Genome-wide analyses identify novel risk loci for cluster headache in Han Chinese residing in Taiwan
Source: J Headache Pain. 2022 Nov 21;23(1):147. doi: 10.1186/s10194-022-01517-6 (PMC9677903; doi:10.1186/s10194-022-01517-6)

Supplemental Figure 1. Quantile-quantile plot of the GWAS results of cluster headache.

The horizontal axis shows -log10 p values expected under the null distribution. The vertical axis shows observed -log10 p values.

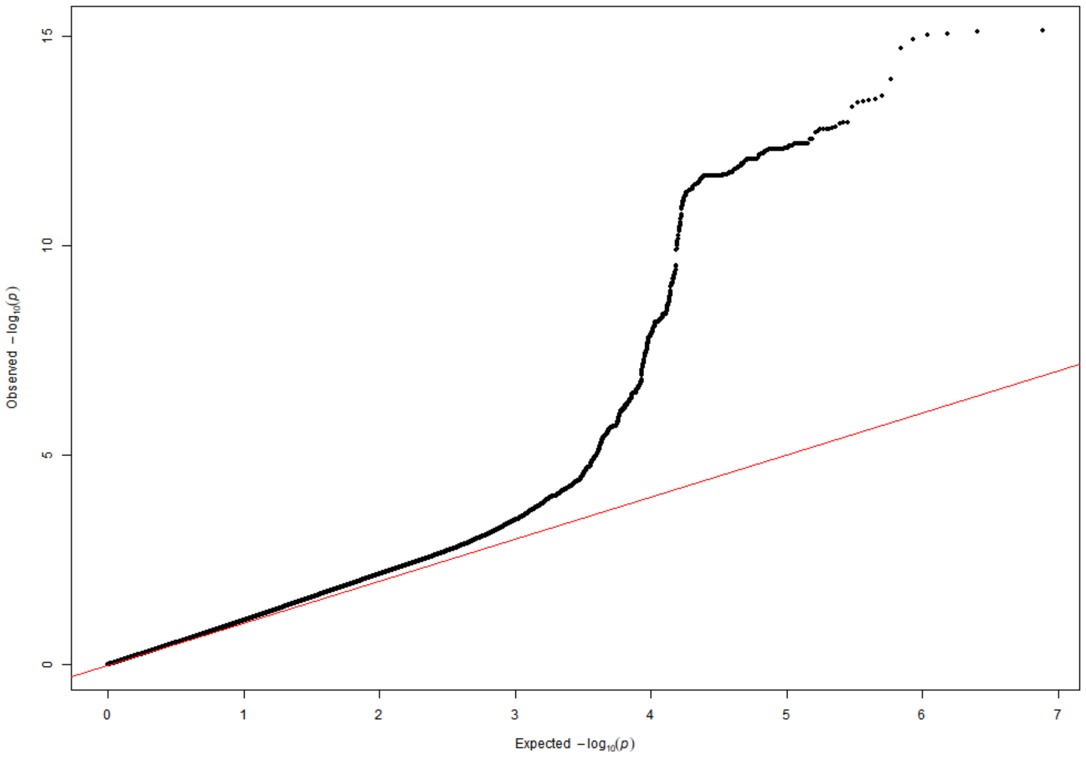

Supplement: Supplementary file 2 — Additional file 2: Supplemental Figure 1. Quantile-quantile plot of the GWAS results of cluster headache. [file 10194_2022_1517_MOESM2_ESM.pdf]
