# Supplementary material for: Genome-wide analyses identify novel risk loci for cluster headache in Han Chinese residing in Taiwan
Source: J Headache Pain. 2022 Nov 21;23(1):147. doi: 10.1186/s10194-022-01517-6 (PMC9677903; doi:10.1186/s10194-022-01517-6)

Supplemental Figure 2. Principal component analysis (PCA) plot.

The horizontal and vertical axes are the first and second dimensions from principal component analysis based on the cluster headache GWAS samples. Blue dots indicate controls and red dots indicate patients.

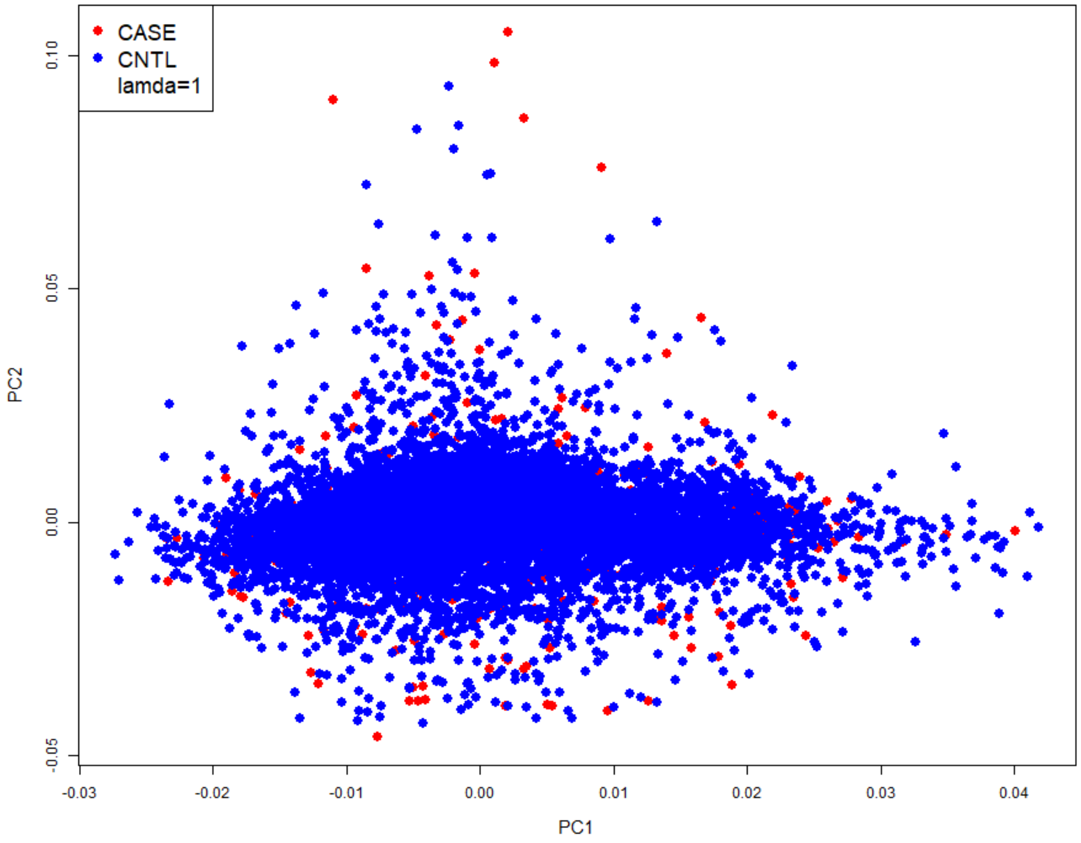

Supplement: Supplementary file 3 — Additional file 3: Supplemental Figure 2. Principal component analysis (PCA) plot. The horizontal and vertical axes are the first and second dimensions from principal component analysis based on the cluster headache GWAS samples. Blue dots indicate controls and red dots indicate patients. [file 10194_2022_1517_MOESM3_ESM.pdf]
