# Supplementary material for: Genome-wide analyses identify novel risk loci for cluster headache in Han Chinese residing in Taiwan
Source: J Headache Pain. 2022 Nov 21;23(1):147. doi: 10.1186/s10194-022-01517-6 (PMC9677903; doi:10.1186/s10194-022-01517-6)

Supplemental Figure 3: Manhattan plot of the discovery cohort (A) and replication cohort (B).

The horizontal axis shows the chromosomal position, and the vertical axis shows the significance of tested markers. The threshold for genome wide significance ($p < 5 \times 10^{-8}$) is indicated by a red dash line.

(A)

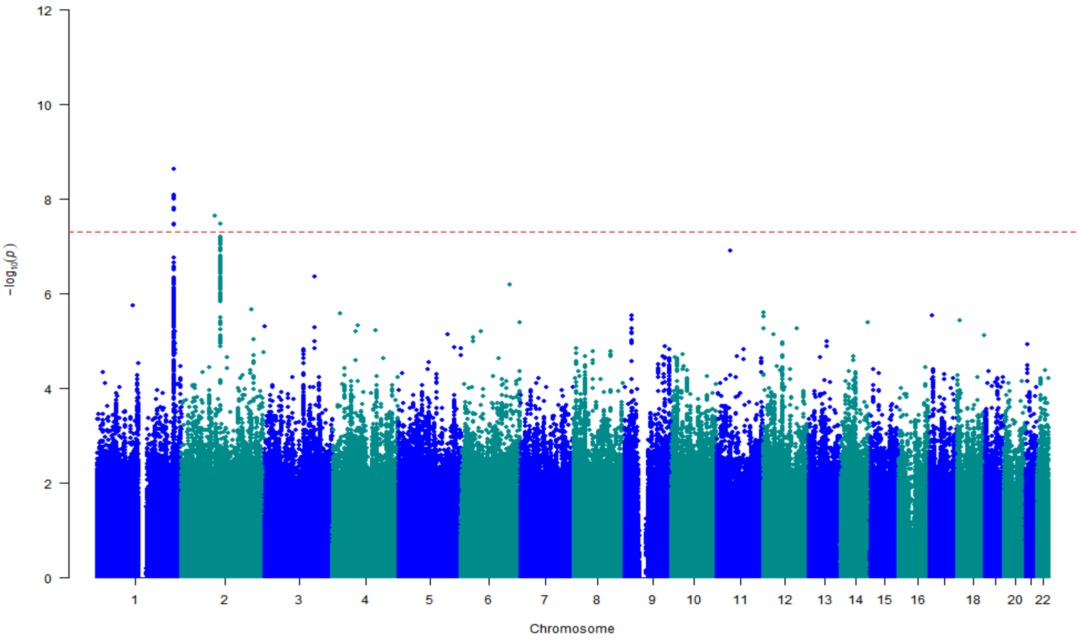

(B)

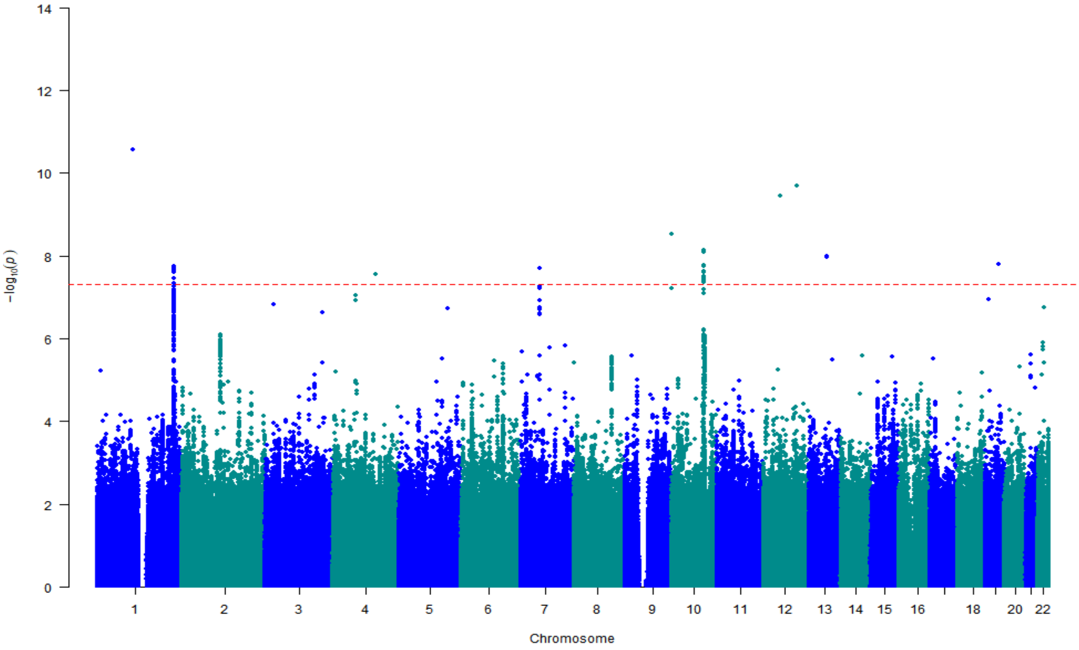

Supplement: Supplementary file 4 — Additional file 4: Supplemental Figure 3. Manhattan plot of the discovery cohort (A) and replication cohort (B). [file 10194_2022_1517_MOESM4_ESM.pdf]
