# Supplementary material for: Genome-wide analyses identify novel risk loci for cluster headache in Han Chinese residing in Taiwan
Source: J Headache Pain. 2022 Nov 21;23(1):147. doi: 10.1186/s10194-022-01517-6 (PMC9677903; doi:10.1186/s10194-022-01517-6)

Supplemental Figure 4. Q-Q plot of the gene-based test computed by MAGMA. The horizontal axis shows -log10 p values expected under the null distribution. The vertical axis shows observed -log10 p values.

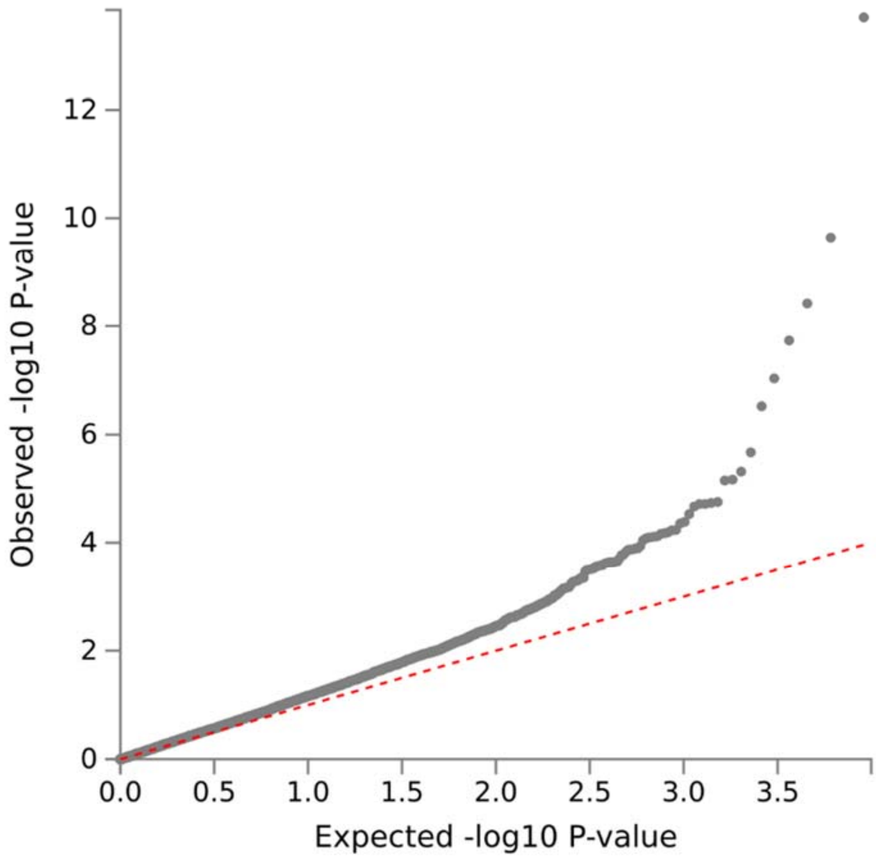

Supplement: Supplementary file 5 — Additional file 5: Supplemental Figure 4. Q-Q plot of the gene-based test computed by MAGMA. The horizontal axis shows -log10 p values expected under the null distribution. The vertical axis shows observed -log10 p values. [file 10194_2022_1517_MOESM5_ESM.pdf]
