# Supplementary material for: Genome-wide analyses identify novel risk loci for cluster headache in Han Chinese residing in Taiwan
Source: J Headache Pain. 2022 Nov 21;23(1):147. doi: 10.1186/s10194-022-01517-6 (PMC9677903; doi:10.1186/s10194-022-01517-6)
Supplement: Supplementary file 6 — Additional file 6: Supplemental Table 2. Meta-analysis of previous studies and the current study for previously reported cluster-associated loci. [file 10194_2022_1517_MOESM6_ESM.docx]

**Supplemental Table 2.** **Meta-analysis of previous studies and the current study for previously reported cluster-associated loci.**

| **Marker Name** | **A1** | **A2** | **Sample size** | **Zscore** | **P-value** | **Direction** | **HetISq** | **HetChiSq** | **HetDf** | **HetPVal** |
| --- | --- | --- | --- | --- | --- | --- | --- | --- | --- | --- |
| rs2499799 | T | C | 12,732 | 2.878 | 0.004001 | ++?? | 96.5 | 28.655 | 1 | 8.65E-08 |
| rs12121134 | T | C | 18,727 | 2.793 | 0.00523 | -??+ | 96.1 | 25.63 | 1 | 4.14E-07 |
| rs2653349 | A | G | 14,227 | -1.643 | 0.1004 | --++ | 73.5 | 11.307 | 3 | 0.01018 |
| rs4519530 | T | C | 18,694 | -10.708 | 9.30E-27 | -??- | 64.6 | 2.822 | 1 | 0.093 |
| rs6541998 | T | C | 12,694 | -7.799 | 6.23E-15 | --?? | 91.2 | 11.363 | 1 | 0.0007492 |
| rs6687758 | A | G | 17,011 | -3.159 | 0.001582 | -??- | 77.1 | 4.358 | 1 | 0.03684 |
| rs11579212 | A | C | 12,814 | -1.697 | 0.08967 | +-?? | 97.3 | 36.959 | 1 | 1.21E-09 |
| rs12649507 | A | G | 13,975 | 3.284 | 0.001022 | +++? | 61.6 | 5.206 | 2 | 0.07405 |
| rs1126671 | T | C | 13,176 | 3.182 | 0.001462 | +++? | 38.6 | 3.255 | 2 | 0.1964 |
| rs11153082 | A | G | 18,761 | -6.51 | 7.54E-11 | -??- | 67.9 | 3.113 | 1 | 0.07768 |
| rs9386670 | A | C | 16,991 | 6.095 | 1.09E-09 | +??+ | 83.1 | 5.902 | 1 | 0.01512 |
| rs3800539 | A | G | 10,580 | 1.518 | 0.129 | +??+ | 0 | 0.014 | 1 | 0.9043 |
| rs10498801 | A | G | 13,050 | -0.951 | 0.3415 | -+?+ | 77.2 | 8.79 | 2 | 0.01234 |
| rs12668955 | A | G | 13,313 | 3.759 | 0.00017 | +++? | 92.5 | 26.754 | 2 | 1.55E-06 |
| rs8192440 | A | G | 11,882 | -1.673 | 0.09438 | -?-? | 81.5 | 5.403 | 1 | 0.02011 |

**Direction** (direction of effect): +(beta>0), -(beta<0), ? (no data),

**HetISq**: I^2^ Heterogeneity statistic

**HetChiSq:** Heterogeneity chi-square statistic;

**HetDf** : Heterogeneity degrees of freedom (HetDf>0，HetDf=0 that only for only one cohort the meta-analysis has been performed, hence this is by definition not a meta-analysis result;

HetPVal: P value of Heterogeneity.
