# Supplementary figures and images for: Genome-wide analyses identify novel risk loci for cluster headache in Han Chinese residing in Taiwan
Source: J Headache Pain. 2022 Nov 21;23(1):147. doi: 10.1186/s10194-022-01517-6 (PMC9677903; doi:10.1186/s10194-022-01517-6)

**Supplemental Figure 5. eQTL analysis by MetaXcan**

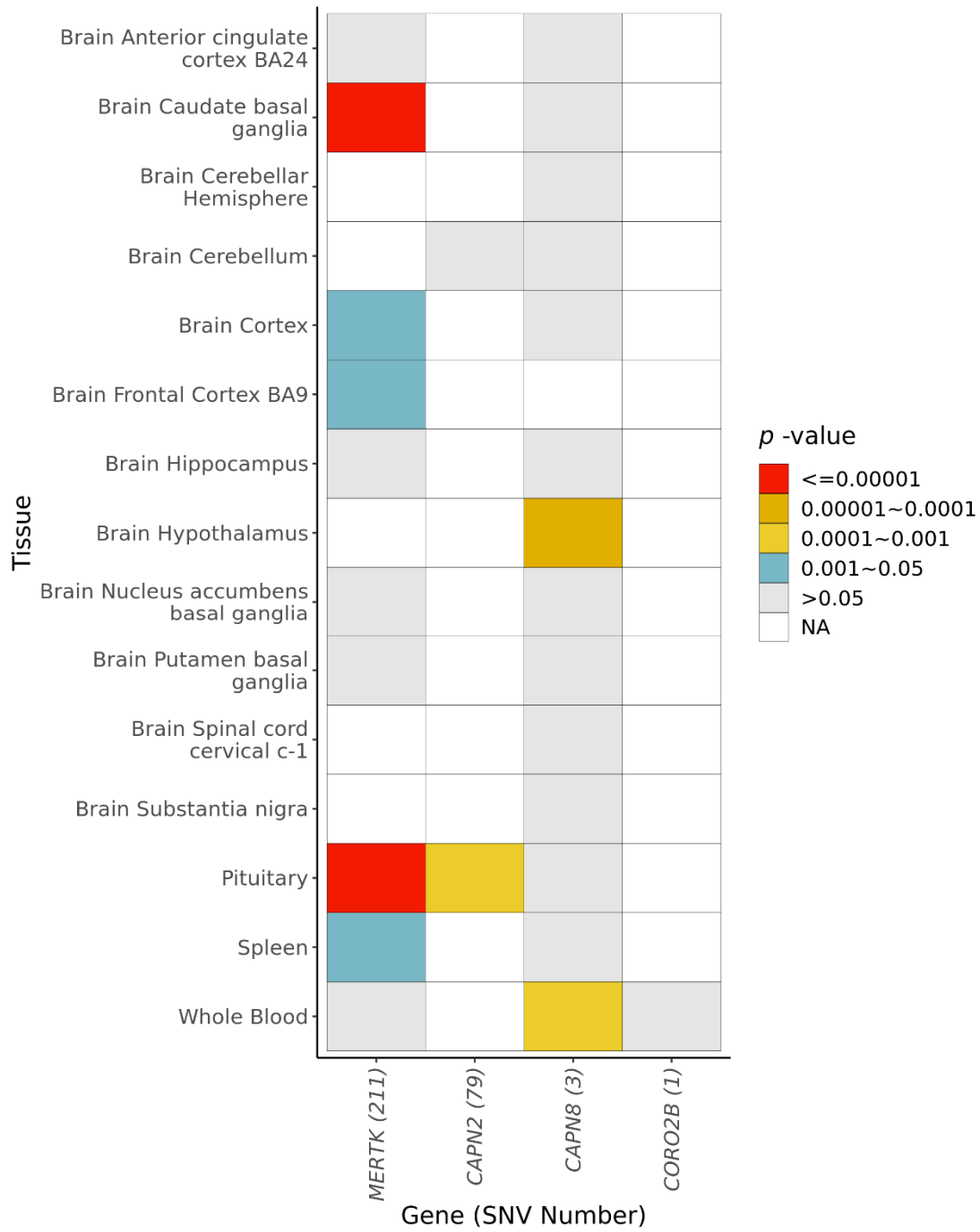

Supplement: Supplementary file 7 — Additional file 7: Supplemental Figure 5. eQTL analysis by MetaXcan [file 10194_2022_1517_MOESM7_ESM.pdf]
