# Supplementary material for: Genome-wide analyses identify novel risk loci for cluster headache in Han Chinese residing in Taiwan
Source: J Headache Pain. 2022 Nov 21;23(1):147. doi: 10.1186/s10194-022-01517-6 (PMC9677903; doi:10.1186/s10194-022-01517-6)
Supplement: Supplementary file 8 — Additional file 8: Supplemental Table 3. GIGSEA Biological Pathway Enrichment in brain tissues. The significant level of empirical P-value was 0.05 and the BayesFactor was 100. Top significantly associated pathways in these tissues that may be relevant to cluster headache were listed in the table. UsedGenes indicates number of gene used in the enrichment estimation of the GIGSEA model. [file 10194_2022_1517_MOESM8_ESM.docx]

**Supplemental Table 3.** **GIGSEA Biological Pathway Enrichment in brain tissues.** The significant level of empirical *P*-value was 0.05 and the BayesFactor was 100. Top significantly associated pathways in these tissues that may be relevant to cluster headache were listed in the table. UsedGenes indicates number of gene used in the enrichment estimation of the GIGSEA model.

| **Category** | **TERM** | **Used**  **Genes** | **Observed**  **Correlation** | **Empirical**  **P-value** | **Bayes Factor** | **Tissue** | **ID** |
| --- | --- | --- | --- | --- | --- | --- | --- |
| synaptic transmission | GOBP_SYNAPTIC_VESICLE_TRANSPORT | 13 | -0.08678 | 0.00039 | 181 | Pituitary | GO:0048489 |
| synaptic transmission | GOBP_SYNAPTIC_VESICLE_LOCALIZATION | 16 | -0.08370 | 0.00051 | 150 | Pituitary | GO:0097479 |
| synaptic transmission | GOBP_SYNAPTIC_VESICLE_CYTOSKELETAL_TRANSPORT | 6 | -0.08340 | 0.00052 | 148 | Pituitary | GO:0099514 |
| synaptic transmission | GOBP_SYNAPTIC_TRANSMISSION_GLUTAMATERGIC | 9 | 0.08438 | 0.00139 | 486 | Brain Hippocampus | GO:0035249 |
| synaptic transmission | REACTOME_PRESYNAPTIC_FUNCTION_OF_KAINATE_RECEPTORS | 6 | 0.09199 | 0.00213 | 504 | Brain Putamen basal ganglia | R-HSA-500657 |
| synaptic transmission | GOBP_NEGATIVE_REGULATION_OF_SYNAPTIC_TRANSMISSION | 8 | 0.07092 | 0.00404 | 192 | Brain Hippocampus | GO:0050805 |
| synaptic transmission | GOBP_CHEMICAL_SYNAPTIC_TRANSMISSION_POSTSYNAPTIC | 14 | 0.06911 | 0.00465 | 166 | Brain Hippocampus | GO:0099565 |
| synaptic transmission | GOBP_REGULATION_OF_POSTSYNAPTIC_MEMBRANE_POTENTIAL | 16 | 0.06672 | 0.00562 | 135 | Brain Hippocampus | GO:0060078 |
| synaptic transmission | REACTOME_NEUROTRANSMITTER_RECEPTORS_AND_POSTSYNAPTIC_SIGNAL_TRANSMISSION | 41 | 0.07679 | 0.00636 | 281 | Brain Putamen basal ganglia | R-HSA-112314 |
| transmembrane protein kinase activity | REACTOME_DISORDERS_OF_TRANSMEMBRANE_TRANSPORTERS | 32 | -0.06833 | 0.01078 | 170 | Brain Putamen basal ganglia | R-HSA-5619115 |
| transmembrane protein kinase activity | GOMF_TRANSMEMBRANE_RECEPTOR_PROTEIN_TYROSINE_KINASE_ACTIVITY | 9 | -0.08378 | 0.00080 | 529 | Pituitary | GO:0004714 |
| transmembrane protein kinase activity | GOMF_TRANSMEMBRANE_RECEPTOR_PROTEIN_KINASE_ACTIVITY | 11 | -0.07100 | 0.00234 | 204 | Pituitary | GO:0019199 |
| transmembrane protein kinase activity | GOMF_PRIMARY_ACTIVE_TRANSMEMBRANE_TRANSPORTER_ACTIVITY | 12 | 0.06597 | 0.00381 | 111 | Pituitary | GO:0015399 |
| transmembrane protein kinase activity | GOBP_PROTEIN_TRANSMEMBRANE_IMPORT_INTO_INTRACELLULAR_ORGANELLE | 9 | -0.06560 | 0.00608 | 123 | Brain Hippocampus | GO:0044743 |
| transmembrane protein kinase activity | GOBP_PROTEIN_TRANSMEMBRANE_TRANSPORT | 13 | -0.06349 | 0.00714 | 102 | Brain Hippocampus | GO:0071806 |
| Immue responses | KEGG_INTESTINAL_IMMUNE_NETWORK_FOR_IGA_PRODUCTION | 9 | -0.14358 | 0.00171 | 115 | Brain Putamen basal ganglia | hsa04672 |
| Immue responses | KEGG_INTESTINAL_IMMUNE_NETWORK_FOR_IGA_PRODUCTION | 14 | -0.12357 | 0.00294 | 29739 | Brain Caudate basal ganglia | hsa04672 |
| Immue responses | KEGG_INTESTINAL_IMMUNE_NETWORK_FOR_IGA_PRODUCTION | 19 | -0.09624 | 0.00536 | 183 | Brain Cerebellum | hsa04672 |
| Immue responses | KEGG_INTESTINAL_IMMUNE_NETWORK_FOR_IGA_PRODUCTION | 10 | -0.14780 | 0.00612 | 2404 | Brain Amygdala | hsa04672 |
| Immue responses | KEGG_AUTOIMMUNE_THYROID_DISEASE | 12 | -0.12419 | 0.01451 | 673 | Brain Amygdala | hsa05320 |
| Immue responses | KEGG_AUTOIMMUNE_THYROID_DISEASE | 14 | -0.09010 | 0.01612 | 2419 | Brain Caudate basal ganglia | hsa05320 |
| Immue responses | GOBP_ADAPTIVE_IMMUNE_RESPONSE_BASED_ON_SOMATIC_RECOMBINATION_OF_IMMUNE_RECEPTORS_BUILT_FROM_IMMUNOGLOBULIN_SUPERFAMILY_DOMAINS | 38 | -0.08928 | 0.00093 | 654 | Brain Hippocampus | GO:0002460 |
| Immue responses | GOBP_IMMUNOGLOBULIN_PRODUCTION_INVOLVED_IN_IMMUNOGLOBULIN_MEDIATED_IMMUNE_RESPONSE | 6 | -0.06501 | 0.00636 | 117 | Brain Hippocampus | GO:0002381 |
| Mitochondrial function | GOBP_PROTEIN_IMPORT_INTO_MITOCHONDRIAL_MATRIX | 5 | -0.08534 | 0.00128 | 517 | Brain Hippocampus | GO:0030150 |
| Mitochondrial function | GOBP_MITOCHONDRIAL_ELECTRON_TRANSPORT_NADH_TO_UBIQUINONE | 11 | -0.07505 | 0.00289 | 264 | Brain Hippocampus | GO:0006120 |
