# Supplementary material for: Genome-wide analyses identify novel risk loci for cluster headache in Han Chinese residing in Taiwan
Source: J Headache Pain. 2022 Nov 21;23(1):147. doi: 10.1186/s10194-022-01517-6 (PMC9677903; doi:10.1186/s10194-022-01517-6)
Supplement: Supplementary file 9 — Additional file 9: Supplemental Figure 6. GIGSEA pathway analysis of the genes relevant to cluster headache GWAS variants. [file 10194_2022_1517_MOESM9_ESM.pdf]

Supplemental Figure 6. GIGSEA pathway analysis of the genes relevant to cluster headache GWAS variants.

We employed the weighted linear regression model of the GIGSEA with empirical p-values incorporating 1,000 permutations for (A-C) Gene Ontology (GO) ((A)GOBP (B)GOCC (C)GOMF), (D) Kyoto Encyclopedia of Genes and Genomes (KEGG), and (E) Reactome (REAC). The Bayes factor was used to correct the multiple hypothesis testing.

A

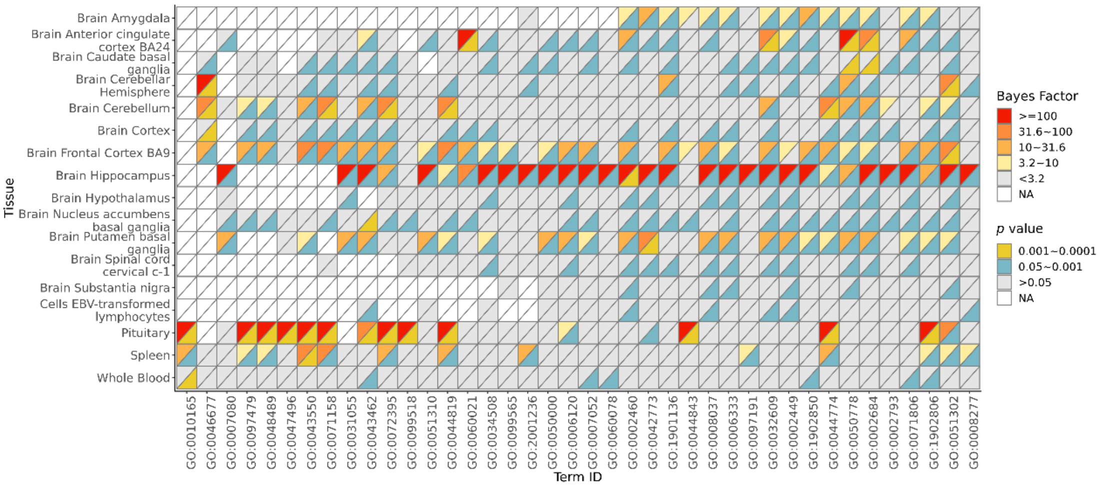

B

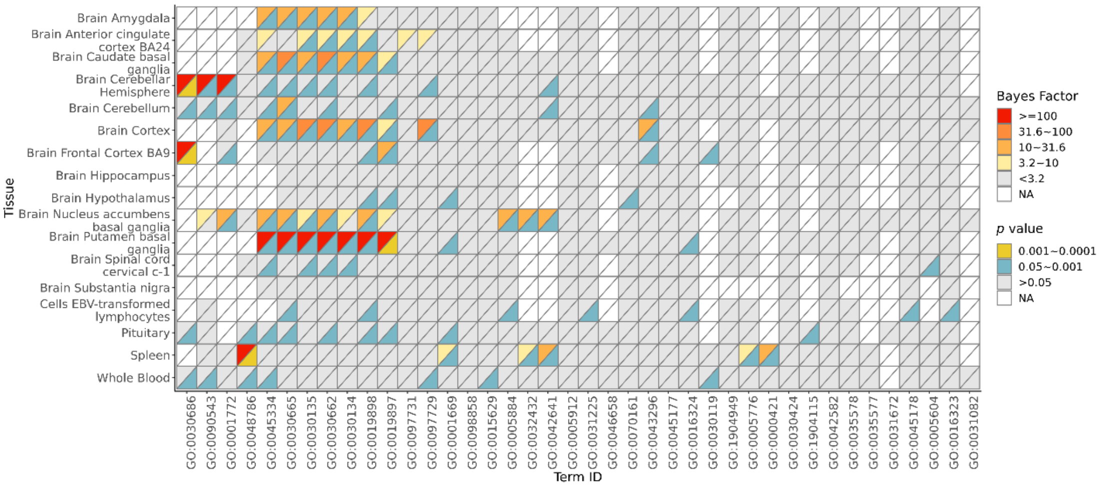

C

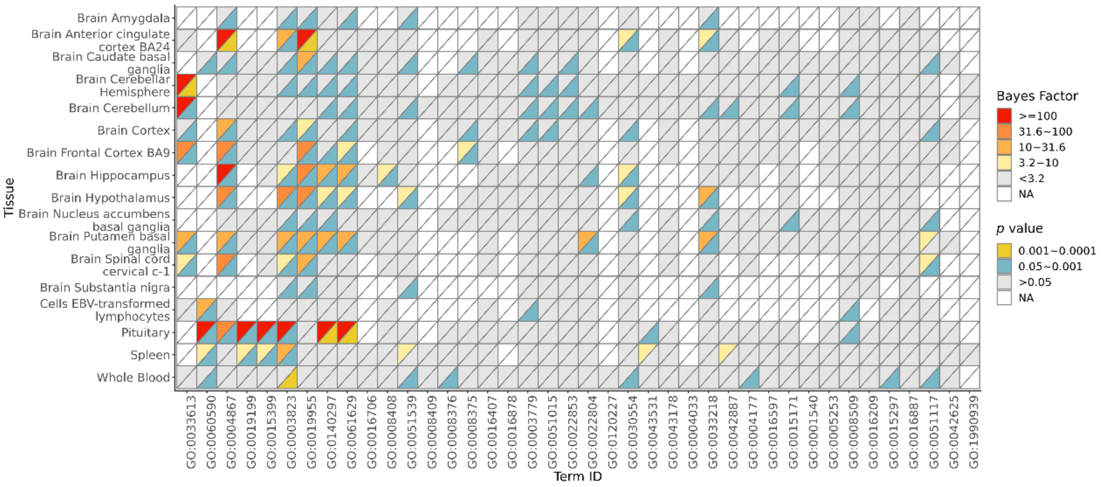

Brain Amygdala
Brain Anterior cingulate cortex BA24
Brain Caudate basal ganglia
Brain Cerebellar Hemisphere
Brain Cerebellum
Brain Cortex
Brain Frontal Cortex BA9
Brain Hippocampus
Brain Hypothalamus
Brain Nucleus accumbens basal ganglia
Brain Putamen basal ganglia
Brain Spinal cord cervical c-1
Brain Substantia nigra
Cells EBV-transformed lymphocytes
Pituitary
Spleen
Whole Blood
Tissue
GO:0033613
GO:0060590
GO:0004867
GO:0019199
GO:0015399
GO:0003823
GO:0019955
GO:0140297
GO:0061629
GO:0016706
GO:0008408
GO:0051539
GO:0008409
GO:0008376
GO:0008375
GO:0016407
GO:0016878
GO:0003779
GO:0051015
GO:0022853
GO:0022804
GO:0120227
GO:0030554
GO:0043531
GO:0043178
GO:0004033
GO:0033218
GO:0042887
GO:0004177
GO:0016597
GO:0015171
GO:0001540
GO:0005253
GO:0008509
GO:0016209
GO:0015297
GO:0016887
GO:0051117
GO:0042625
GO:1990939
Term ID
Bayes Factor
>=100
31.6~100
10~31.6
3.2~10
<3.2
NA
p value
0.001~0.0001
0.05~0.001
>0.05
NA

D

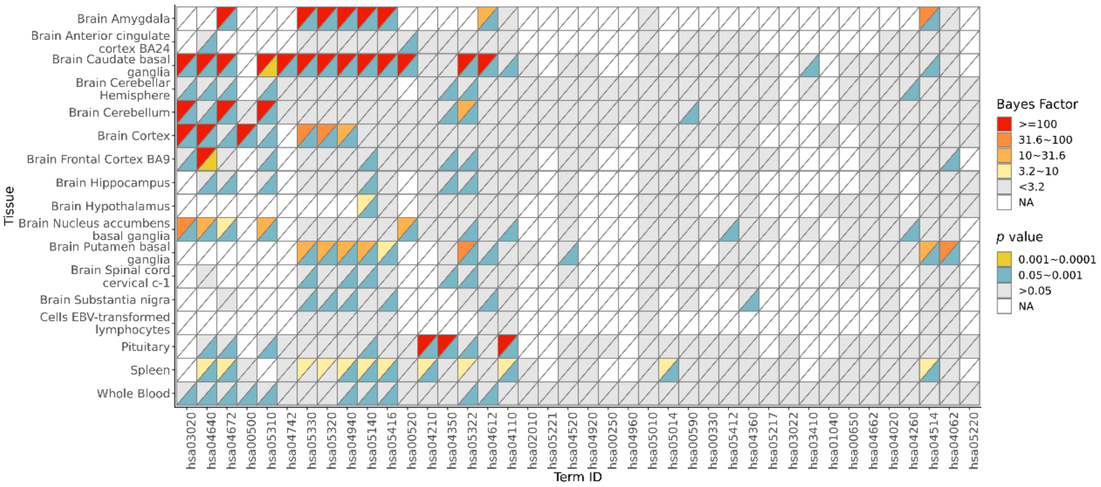

Brain Amygdala
Brain Anterior cingulate cortex BA24
Brain Caudate basal ganglia
Brain Cerebellar Hemisphere
Brain Cerebellum
Brain Cortex
Brain Frontal Cortex BA9
Brain Hippocampus
Brain Hypothalamus
Brain Nucleus accumbens basal ganglia
Brain Putamen basal ganglia
Brain Spinal cord cervical c-1
Brain Substantia nigra
Cells EBV-transformed lymphocytes
Pituitary
Spleen
Whole Blood
Tissue
hsa03020
hsa04640
hsa04672
hsa00500
hsa05310
hsa04742
hsa05330
hsa05320
hsa04940
hsa05140
hsa05416
hsa00520
hsa04210
hsa04350
hsa05322
hsa04612
hsa04110
hsa02010
hsa05221
hsa04520
hsa04920
hsa00250
hsa04960
hsa05010
hsa05014
hsa00590
hsa00330
hsa05412
hsa04360
hsa05217
hsa03022
hsa03410
hsa01040
hsa00650
hsa04662
hsa04020
hsa04260
hsa04514
hsa04062
hsa05220
Term ID
Bayes Factor
>=100
31.6~100
10~31.6
3.2~10
<3.2
NA
p value
0.001~0.0001
0.05~0.001
>0.05
NA

E

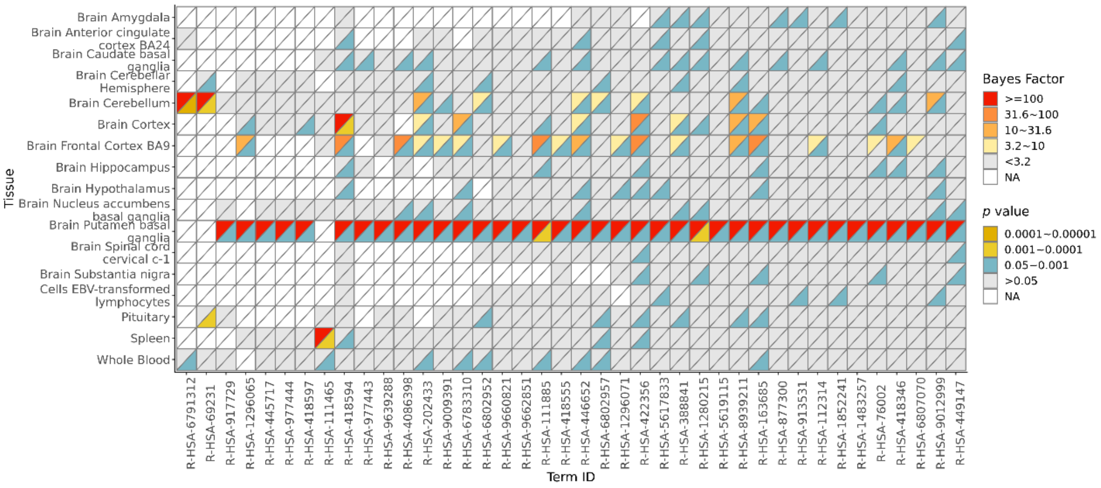

Brain Amygdala
Brain Anterior cingulate cortex BA24
Brain Caudate basal ganglia
Brain Cerebellar Hemisphere
Brain Cerebellum
Brain Cortex
Brain Frontal Cortex BA9
Brain Hippocampus
Brain Hypothalamus
Brain Nucleus accumbens basal ganglia
Brain Putamen basal ganglia
Brain Spinal cord cervical c-1
Brain Substantia nigra
Cells EBV-transformed lymphocytes
Pituitary
Spleen
Whole Blood
Tissue
R-HSA-6791312
R-HSA-69231
R-HSA-917729
R-HSA-1296065
R-HSA-445717
R-HSA-977444
R-HSA-418597
R-HSA-111465
R-HSA-418594
R-HSA-977443
R-HSA-9639288
R-HSA-4086398
R-HSA-202433
R-HSA-9009391
R-HSA-6783310
R-HSA-6802952
R-HSA-9660821
R-HSA-9662851
R-HSA-111885
R-HSA-418555
R-HSA-446652
R-HSA-6802957
R-HSA-1296071
R-HSA-422356
R-HSA-5617833
R-HSA-388841
R-HSA-1280215
R-HSA-5619115
R-HSA-8939211
R-HSA-163685
R-HSA-877300
R-HSA-913531
R-HSA-112314
R-HSA-1852241
R-HSA-1483257
R-HSA-76002
R-HSA-418346
R-HSA-6807070
R-HSA-9012999
R-HSA-449147
Term ID
Bayes Factor
>=100
31.6~100
10~31.6
3.2~10
<3.2
NA
p value
0.0001~0.00001
0.001~0.0001
0.05~0.001
>0.05
NA
